# Wild leafy vegetable use and knowledge across multiple sites in Morocco: a case study for transmission of local knowledge?

**DOI:** 10.1186/1746-4269-10-34

**Published:** 2014-04-04

**Authors:** Bronwen Powell, Abderrahim Ouarghidi, Timothy Johns, Mohamed Ibn Tattou, Pablo Eyzaguirre

**Affiliations:** 1 Centre for International Forestry Research, Bogor, Indonesia; 2 School of Dietetics and Human Nutrition, McGill University, Montreal, Canada; 3 Faculty of Science, University of Cadi Ayyad, Marrakech, Morocco; 4 Département de Botanique et Ecologie Végétale, Institut Scientifique de Rabat (ISR), University Mohammed V - Agdal, Rabat, Morocco; 5 Bioversity International, Rome, Italy

**Keywords:** Wild food, Leafy vegetables, Traditional food, Traditional food system, Local knowledge, Ethnobotany, Nomenclature, Nutrition

## Abstract

**Background:**

There are few publications on the use and diversity of wild leafy vegetables (WLVs) in Morocco. In order to address this gap, we conducted ethnobotanical field work in Taounate, Azilal and El House regions.

**Methods:**

Ethnobotanical collections, free listing, qualitative interviews and a 7 day food frequency questionnaire.

**Results:**

More than 30 species in 23 genera of WLV were identified. Of these 4 had not previously recorded as WLVs used in Morocco in the literature. WLVs were used by 84% of households surveyed in Taounate (N = 61, in March 2005), and were used up to 4 times a week. Qualitative data revealed both positive and negative perceptions of WLVs and detailed knowledge about preparation among women. The greatest diversity of WLV knowledge and use was in the Rif Mountains (Taounate). There was significant variation in nomenclature and salience of WLVs, not only between regions, but also between villages in the same region. Within the same region (or even village) different local names were used for a given species or genus, and different species were identified by the same local name (including species from different botanical families). Data showed greater overlap in knowledge among villages using the same market.

**Conclusion:**

We believe the results suggest that markets are important sites for WLV knowledge transmission.

## Introduction

Until recently, wild food use in Morocco has been significantly under-reported in the literature. In a recent publication, Nassif and Tanji [1] list almost 80 species used as wild vegetables in Morocco (see table one which lists all wild vegetables, as well as fruits, seeds and other wild foods), identified from francophone grey literature sources and their own anecdotal observation in the field. They note the need for systematic studies of wild foods in Morocco, similar to those that have been carried out in other regions.

Before Nassif and Tanji’s 2013 [1] publication only a few wild vegetables species had been list in Morocco in Tanji and Nassif 1995 [2] and Hadjichambis et al. 2008 [3]. The gap in scientific documentation may be explained by the seasonal or geographic variation in use, the fact that much of the botanical and ethnobotanical research conducted in Morocco has been overseen by male researchers and wild vegetables are a women’s domain, and the fact that most ethnobotanical work in Morocco focuses on medicinal and aromatic plants. In an effort to further scientific documentation on this topic, this paper documents the distribution and use of wild vegetables across multiple sites in Taounate, Azilal, and El Haouz, using botanical identification and voucher collection. Herein we examine geographic differences in use, diversity, salience and ethnobotanical nomenclature of WLVs. We conclude by examining potential drivers of geographic variation in nomenclature and use.

Wild vegetables are an important component of traditional food systems around the world [4,5]. Wild vegetables are a distinct cultural domain in the knowledge systems of many communities. They are called “Liakra” in Arberesh, Southern Italy [6], “Michicha” by the Shambaa in Tanzania [7,8] and “Rau Dai” in Vietnam [9]. Likewise, in Morocco they are known as *Bakola* or *Khobiza,* terms which also refer to the best known WLV *Malva* spp.. In this paper we use the term wild leafy vegetables (WLVs) because almost all wild vegetables in Morocco are leafy; however, we also include species used for their stems or other vegetative parts as they are also considered part of the cultural domain *Baloka* or *Khobiza*. Aromatic plants used as seasoning or in tea are not included.

WLVs are essential to the nutrition and food security of people around the world. WLVs add diversity to the diet; making diets healthier and more interesting [9-11]. Studies have shown the significant contribution of WLVs to micronutrient content of local diets in developing countries [9,12]. WLVs can be important, not just in times of food scarcity (drought) but throughout the year [10,13,14]. In many cases, WLVs are especially important to socio-economically vulnerable groups [10] and indigenous populations forced to live on marginal lands for social and political reasons [15]. In such groups WLVs can decrease people’s dependence on cash-purchased market foods and provide income for those with limited access to land for cultivation of crops [7,15].

The use of WLVs is suggested to be declining around the world [16], a shift associated with a general nutrition and dietary transition [17]. Chweya and Eyzaguirre [16] cite numerous causes for declining use of traditional and wild vegetables in Africa including: decreased availability due to biodiversity loss and change in agricultural practise, government and development policies that ignore WLVs; loss of knowledge needed for gathering and preparation; and a general loss of cultural value for WLVs. Highlighting that at-risk groups suffer the most from decreasing availability of WLVs, are studies showing that it is poor women who first notice declines in availability and diversity of WLVs species [15,18]. Socio economic and cultural devaluation is a frequently noted factor in the declining use of WLVs around the world [16,19,20]. In Nepal, nettles (*Urtica* spp.) are eaten by monks living a life of frugality and thus are considered to be part of only the most meagre diet [15]. In Spain WLVs are seen as  old-fashioned’ and time-consuming to collect, despite the fact that many people like the taste and enjoy gathering them [19]. WLVs are an integral part of the biodiversity of agro-ecosystems that enhances resilience of the traditional food system, and provides local and culturally appropriate options to help mitigate both chronic, diet and over-nutrition related diseases and micronutrient deficiency concurrently [4,5,21].

This paper reports the results of a free listing exercise for WLVs with women, as well as the botanical identification of many of these species. Data on the frequency of WLV consumption, obtained from a 7 day food frequency questionnaire are reported, followed by qualitative data on perceptions and knowledge of WLVs. The paper also includes an examination of geographic variation in use, salience and nomenclature and a discussion of the potential role of markets in the transmission of knowledge about WLVs. The paper concludes with thoughts on the role of WLV in traditional food systems and nutrition, and the potential role of markets in nutrition education and public health nutrition campaigns in Morocco.

## Methods

### Study sites

Morocco has one of the highest level of biodiversity in the Mediterranean [22] and is also a country facing significant issues in overcoming malnutrition. The country is undergoing a nutrition transition [23,24]: 36.6% of women are estimated to be overweight or obese [25] while micronutrient deficiencies (especially vitamin A and iron) remain a problem [26]. The national prevalence of vitamin A deficiency (serum retinol < 20 μg/dl) in children under 5 years old was estimated to be 35.1% in 2007 [26] and stunting remains a problem, with 23.6% of rural children stunted (Height for age Z score -2 S.D.) [25]. The country now faces a double burden of disease; with chronic nutrition-related disease and micronutrient deficiency and infectious disease simultaneously problematic. Because WLVs were historically not well documented in the country, efforts to address both micronutrient deficiency and chronic nutrition-related diseases have made little mention of the potential benefits of them and other traditional foods. As is the case elsewhere in the world, the high number of species used, geographical variation in use and nomenclature and lack of nutritional composition data, has surely contributed to the lack of incorporation of WLVs into nutritional and agricultural research and education [16].

**Figure 1:**
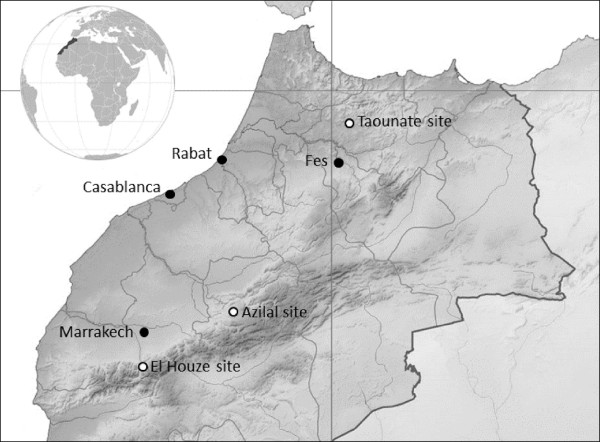
Map of northern Morocco showing major cities and location of research sites.

This paper presents data from 3 ecologically distinct regions: Taounate, Azilal, and El Haouz provinces (Figure 1). Taounate is located in the Rif Mountains about 80 km north of Fes in northern Morocco. The main livelihood activity in the area is agriculture; however, although no households involved in the research reported direct participation in cannabis cultivation, many in the area earn some or most of their annual income through involvement in cannabis trade. In Taounate work was conducted primarily in three villages: Izara (IZR), Sidi Sinoun (SSN) and Chachia (CHC). Additional research was also carried out with roadside vendors in Ait Bouhamou (ABH). Azilal is located 160 km east of Marrakech in the High Atlas Mountains (central-eastern Morocco). The area is considered under-developed with limited potential for agriculture and tourism. Within Azilal, research was conducted in 2 villages: Ibiyane (IBN) and Habliss (HBL). El Haouz province covers much the High Atlas Mountains directly south of Marrakech (central-south Morocco). Work was conducted in the village of Tassa Ouirgen, 65 km south (and a bit west) of Marrakech and adjacent to the Toubkal National Park, the center-piece of tourism in the High Atlas Mountains.

The female head of household or primary cook for every household in each village was identified and approached to be interviewed. Each woman consenting to participate in the research responded to the household food frequency questionnaire (FFQ). Additionally they were also asked to provide a free list of all the wild vegetables they could list. Not all women who agreed to respond to the FFQ were willing or able to give a free list (about half the women who responded to the FFQ provided a free list). In Tassa Ouirgen no FFQ was conducted with the six women who gave free lists. In Tassa Ouirgen the women involved were identified as knowledgeable by the community leaders and each other. Informants for the qualitative information collected were encountered during participant observation, field visits and research activities and were not selected using any specific system. Research was conducted in accordance with the International Society of Ethnobiology (ISE)’s Code of Ethics.

### Contribution to diet and nutrition

A household qualitative food frequency questionnaire (FFQ) was conducted with the female head of the household to record the food items used in the last week (Taounate March 10-24th, 2005, N = 61 and Azilal April 12-20th, 2005, N = 42). The FFQ recorded the number of days out of the last 7 the household had used WLVs, but not the number of different species used (when research began we expected only one or a few species were used).

In order to begin to understand the potential contribution WLVs could make to health and nutrition in Morocco the nutrient compositions of the most common WLVs were examined, drawing from the FAO Food Composition Tables [27], USDA Nutrient Data Base and an extensive literature search for the most recent and most accurate data (there is no Food Composition Table for Morocco).

### Lists of species used in each site

Free listing was conducted with women after they had completed the FFQ (Taounate - Sidi Sinoun N = 13, Izara N = 8, Chachia N = 14; Azilal - Ibiyane N = 10, Habliss N = 10; El Haouz - Tassa Ouirgen N = 6). Informants were asked to free list all the “Wild Leafy Vegetables” or “Plants used with/like *Malva* spp.” they could name, with prompting to only include vegetables (not aromatic or medicinal plants) and wild species (not cultivated ones). After a list of local names was compiled, key-informants were asked to take us to collect as many species as possible in each site. Research was conducted over multiple years (2005–2012) as timing for collection of WLV species is complicated by short and variable flowering times. Collections were made, numbered and dried following standard methodologies. Some species were identified *in situ* by Abderrahim Ouarghidi, who has extensive botanical training and experience in Morocco. Collected vouchers were identified by Bronwen Powell and Abderrahim Ouarghidi (using Petite Flore des Régions Arides du Maroc Occidental by Nègre, 1961 and Nouvelle Flore de l’Algérie by Quezel and Santa, 1962), with confirmation by Ibn Tattou. The vouchers were deposited in the herbarium at Institut Scientifique de Rabat (ISR).

### Qualitative data

Participant observation, unstructured questions and discussion were used to collect qualitative data about WLVs. Information was recorded in field notes. The extent of questions and discussion was left at the discretion of the interviewer and informant, and was intended to gain information about taste, health benefits, accessibility and knowledge/identification of WLVs species.

### Analysis of free lists

Free lists were analyzed using Anthropac, a computer program that specializes in cultural domain analysis and Multi-Dimensional Scaling (MDS) [28]. The MDS graphs of the variation between informants’ Free lists allows for clusters of informants (those with similar responses/most similar knowledge) to be identified.

## Results and discussion

### Species used

In Taounate 29 separate local names were recorded (not including small variations), seven in Azilal, and 12 in El Haouz. We have identified more than 30 species of WLVs used across Morocco. Most vernacular names correspond to the botanical taxonomic division of genus, of which we have identified 23 (Table 1). There remain multiple vernacular names for plants which we have not been able to identify as informants use only the early leaves and often cannot identify the plants once they are mature.

**Table 1:** Species used as WLVs and their local names in 3 different sites in Morocco

**Family**	**Botanical Name**	**Identification**	**Local names**			**Previously reported in literature**
			**Taounate**	**Azilal**	**El Haouz**	
Apiaceae	*Ammi majus* L.	In field, AO 2007 BP2012-13/14/15/63/64/66	TRAYLAN/TRYLANE* (SSN, CHC, ABH) TRYLA/TRYLILA (CHC, IZR)	Not recorded	Not recorded	Bellakhdar 1997 [29], tlaylan, traylal
Apiaceae	*Ferula communis* L.	BP2012-12/31/67	SLILY	Not recorded	WA’MSA	Bellakhdar 1997 [29], boubal
Apiaceae	*Foeniculum* sp. (cf *F. vulgare* Mill.)	In field, AO 2012	Not collected, possibly SLILY	Not recorded	WA’MSA	Bellakhdar 1997 [29], besbes wamssa
Apiaceae	*Ridolfia segetum* Moris	In field, AO 2005 BP2012-45	SLILY	Not recorded	(maybe another var. of WA’MSA)	Bellakhdar 1997 [29] and Tanji and Nassif 1995 [2] in Chaouia and Doukkala, tebch
Asteraceae	*Anacyclus clavatus* (Desf.) Pers. and *Anacyclus radiatus* Loisel.	BP2012-21/40/41/58	KRAI GHORAB (CHC) KHOBIZA (SSN)	Not recorded	Not recorded	Not reported
Asteraceae	*Calendula arvensis* L., *Calendula* cf. *suffruticosa* Vahl and other *Calendula* spp.	In field, AO 2005 BP2012-16/17/32/33/34	NOUR JENA (SSN, IZR)	Not recorded	Available but not eaten	Not reported
Asteraceae	*Carduus tenuiflorus* Curtis.	BP2012-09/10	Maybe also called GERNINA	Maybe also called TAGHDIOUT	TAGHIDIOUT	Not reported
Asteraceae	*Centaurea aspera* L.	BP2012-48/49	FOUWAHA (ABH, IZR)	Not recorded	Not recorded	Tanji and Nassif 1995 [2] in Chaouia, daaga, shefraj, zmamour
Asteraceae	*Glebionis coronaria* (L.) Cass. ex Spach	BP2012-51/57	KRAA, DJEJEA KRAA, RJEL FLOUS (ABH)	Not recorded	Not recorded	Nassif and Tanji 2013 [1], gahwan, kraa djaja, ghadou mlal
Asteraceae	*Leontodon* spp., *Leontodon maroccanus* Ball, *Leontodon saxatilis* Lam.	BP2012-26/27/38/39	HALIOWA (SSN)	Not recorded	Not recorded	Nassif and Tanji 2013 [1] in Marmoucha, tizodia
Asteraceae	*Scolymus maculatus* L. and *Scolymus hispanicus* L. (local names might also refer to *Echinops spinosus* L. and *Carduus* spp.)	In field, AO 2007 BP2012-23/59	GERNINA, JARNIJ (ABH, CHC) SRA (SSN, IZR)	TAGHDIOUT	TAGHDIOUT	Bellakhdar 1997 [29] and Tanji and Nassif 1995 [2], guernina, taghediwt
Asteraceae	*Sonchus oleraceus* L., and p*o*ssibly other *Sonchus* spp.	BP2012-02/03/04	Available but not eaten	Not recorded	ANKRASH AMGHOUD	Bellakhdar 1997 [29] and Tanji and Nassif 1995 [2], Nassif and Tanji 2013 [1], tifaf, tadgarnit
Brassicaceae	*Diplotaxis catholica* (L.) DC. and *Diplotaxis tenuisiliqua* Delile and *Diplotaxis* sp.	In field, AO 2007 BP2012-46/55/56	BOUHAMOU (all but CHC) SHARIAT (CHC)	Not recorded	Available but not eaten	Bellakhdar 1997 [29], kerkaz, cheryat
Brassicaceae	*Nasturtium officinale* R.Br.	In field, AO 2007 BP2012-06	GARNOUNCH	Not recorded	GERNOUNCH (w flws), TKLEM GELSHIEFT (no flws)	Bellakhdar 1997 [29] Nassif and Tanji 2013 [1], gernounej
Brassicaceae	*Sinapis* spp. (possibly *S. arvensis* L.)	In field, AO 2007	BOUHAMOU** (SSN, IZR) SHARIAT (CHC)	Not recorded	Available but not eaten	Nassif and Tanji 2013 [1], bahaou
Brassicaceae	*Sisymbrium officinale* (L.) Scop.	BP2012-20	SHARIAT (CHC)	Not recorded	Not recorded	Nassif and Tanji 2013 [1] in Marmoucha, laihyane
Caryophyllaceae	*Silene vulgaris* (Moench) Garcke	BP2012-07/08	No record	Not recorded	TAGHIRASHT	Bellakhdar 1997 [29] Nassif and Tanji 2013 [1], tighecht, tighighit
Chenopodiaceae	*Beta macrocarpa* Guss.	No identification, based on rosettes and other literature	Possibly SELK	Not recorded	Not recorded	Nassif and Tanji 2013 [1], in Marmoucha, selg, tibidas
Chenopodiaceae	*Chenopodium album* L., also *Chenopodium* spp.	In field, AO 2005 BP2012-24/25	RJEL FLOUS (sometimes FOURA in SSN)	MOUD MAZIR (IBN)	Available but not eaten	Not reported
Geraniaceae	*Erodium moschatum* L’Her. Ex Aiton, *Erodium touchyanum* Delile ex Godr.*, Erodium praecox* (Cav.) Mendonca & Carv.	In field, AO 2007 BP2012-35/36/37/54	KARN KEBISHA/KARN KEBSA (SSN, IZR) MESK AZARA*** (CHC)	Available but not eaten	Available but not eaten	Bellakhdar 1997 [29], hellalt neyreb, lkhellal
Malvaceae	*Malva* spp. (*M. parviflora* L.*, M. nicaeensis* All*., M. neglecta* Wallr., *M. hispanica* L.) (Nomenclature varied from one family to another, usually one vernacular name for the whole generic complex)	In field, AO 2005 BP2012-01/22/28/29/ 47/70	BAKOLA HOURA, BAKOLA HOURIN, KHOBIZA, KHOBIZA GHRAB	TIBI KHOBIZA	TIBI KHOBIZA	Bellakhdar 1997 [29], Tanji and Nassif 1995 [2], khoubbiza, lkhoubiez, tibi, abajir, beqoula
Polygonaceae	*Emex spinosa* (L.) Campd.	BP2012-42/43/44	HOUMIDA (and sometimes SELK)	HOUMIDA	HOUMIDA	Bellakhdar 1997 [29], Tanji and Nassif 1995 [2], lhenzab, hoummedia, tassemount
Polygonaceae	*Rumex pulcher L., Rumex obtusifolius L. and Rumex* spp. (possibly *R. crispus* L.*, R. vesicarius* L.)	In field, AO 2007 BP2012-18/19/65/71	HOUMIDA	ITZ, IBITZ, IBITAZ, TIFILSHOUT	Possibly TIFILSHOUT	Bellakhdar 1997 [29], hoummedia, tassemount
Portulacaceae	*Portulaca oleracea* L.	In field, AO 2005	TRJLA	Not recorded	TAGERLOUCHT	Bellakhdar 1997 [29], Tanji and Nassif 1995 [2], Hadjichambis et al. [3] trejla, agertin, timeqsine

*In IZR Traylane is reported as another name for Trylila.

**No Shariat reported in SSN or IZR and no Bouhamou reported in CHC, indicating likely different names for same plant.

***In SSN and IZR informants say Mesk Azara is the same as or a type of Kam Kebsa.

Of the species/genera we identified there are 4 (over 15% of those we identified) that were not previously recorded as WLVs use in Morocco in the literature, including: *Anacyclus clavatus* (Desf.) Pers. and *Anacyclus radiatus* Loisel, multiple spp. of *Calendula, Carduus tenuiflorus* Curtis., and at least one species of *Chenopodium*. Compared to the vernacular names reported in the literature, at least one of the names we recorded, matched at least one of the names previously recorded, 80 percent of the time. These data provide valuable additional empirical evidence in support of previously published lists.

We also interviewed six vendors selling WLVs on the side of the main road from Taounate to Fes. These six included five men and one woman, all between the ages of 15 and 35 years old from villages near the road. Other vendors along the same section of the road sell fermented milk (lben) and snails. They reported that the types of WLVs they sold included: Sekoum (*Asparagus* spp. from Nassif and Tanji [1]), Khobiza (*Malva* spp.), Rjel Felous (*Chenopodium* sp, or *Glebionis coronaria* (L.) Cass. ex Spach), Trylane (*Ammi majus* L.), Houmida (*Rumex* or *Emex* spp.), Bouhamu (*Diplotaxis* or *Sinapis* spp.).

### Frequency of consumption and potential contribution to nutrition

In our field sites in Morocco all WLV are cooked either as their own side dish (called salad, but cooked) or used as a vegetable in a sauce poured over couscous. We have not identified any wild vegetables that are commonly consumed raw in the household (some may be occasionally eaten raw by children). In Chachia and Sidi Sinoun 78% and 77% of households reported having used WLVs in the past 7 days. Interestingly in Izara, the village with the lowest engagement in agriculture and easiest access to the road, 95% of households reported having used WLVs at least once in the past 7 days. Of the households using WLVs, they were used between one to four times per week, with an average of 1.7 days. Compared to the average 84% of households surveyed in Taounate that had used WLVs in the past week, in Azilal only two households (5%) reported having used WLVs in the past week. It is not possible to determine whether this was due to seasonal differences or differences in use between the two sites.

We attempted to compare the nutrient composition of the most commonly consumed WLVs in Morocco. Nutrient composition data for wild and indigenous species is notoriously poor [8,30]. Although calls for improved data on nutrient composition of wild and traditional foods have done much to stimulate research on this topic across Africa and the Mediterranean [31,32], there remains insufficient data on the content of important nutrients in the most commonly used WLV species in Morocco to evaluate their potential contribution to overcoming nutrient deficiencies. For example, although there was some data for *Malva*, the most common genus, there is a large variation in nutrient compositions cited and no composition data specific to Morocco (e.g. [33]). Moreover, we found no data on the micronutrient content of *Scolymus hispanicus*, one of the common WLVs in Morocco, in any Food Composition Table or in the literature. Further research is clearly needed.

### Qualitative data on perceptions of WLVs

We encountered both very positive and very negative attitudes towards WLVs. However attitudes are increasingly positive in recent years, although it is unclear whether this is only the case in our research sites or across Morocco. In Taounate (2007) informants told us a local proverb: “*Likayakol bakola Amro maysagad chilogha*/If a child eats too much *bakola* when he is small he will not learn to speak (because *bakola* is meant for animals and animals don’t speak)”. Four households in Chachia (2005) showed marked disdain for the use of WLVs and denied they knew any of their names. These same houses did not report use of WLV. Similar denial of knowledge and use was seen in Azilal (Habliss and Ibiyan) where informants insisted that they only used *Malva* spp. and nothing else, these responses were more common when the interviewer did not speak Berber. However in other cases, women were apologetic that their knowledge about WLVs was limited.

More recently in El Haouz, some women told us that people prefer WLVs to cultivated/imported vegetables because they taste better and/ or because they have medicinal properties: that they are “hot” (including Malva spp., Nasturtium officinale (R.Br.) and Ferula communis (L.)). Many people (both recently and less recently) have told us WLVs are good for health in general, but others claim they get a stomach ache or diarrhea when they eat WLVs or that WLVs are bad for diabetes. Interesting it has been mostly men who have reported negative health effects of WLVs, and mostly women reporting positive effects. It is possible that misidentification due to insufficient knowledge could lead to some incidences of gastro-intestinal discomfort or more sever toxicity.

Others reported that WLVs are only used if and when other vegetables are not available. For example in Chachia (2005) we were told that once the Faba beans were ripe, many families would no longer use WLVs. Local women in Tassa Ouirgen (2012) noted that no one in the village dries WLVs but that they thought people used to dry them when there was greater risk of food insecurity, however now that they are  developed’ (with greater market integration) people no longer need to dry WLVs.

Formal education could theoretically influence knowledge and perceptions of WLVs, however very few of the women we interviewed (and very few women in rural Morocco in general) had any formal education, so comparison of those with more and less formal education was not possible. However, there was no evidence of significant differences in knowledge or perceptions due to formal education.

**Figure 2:**
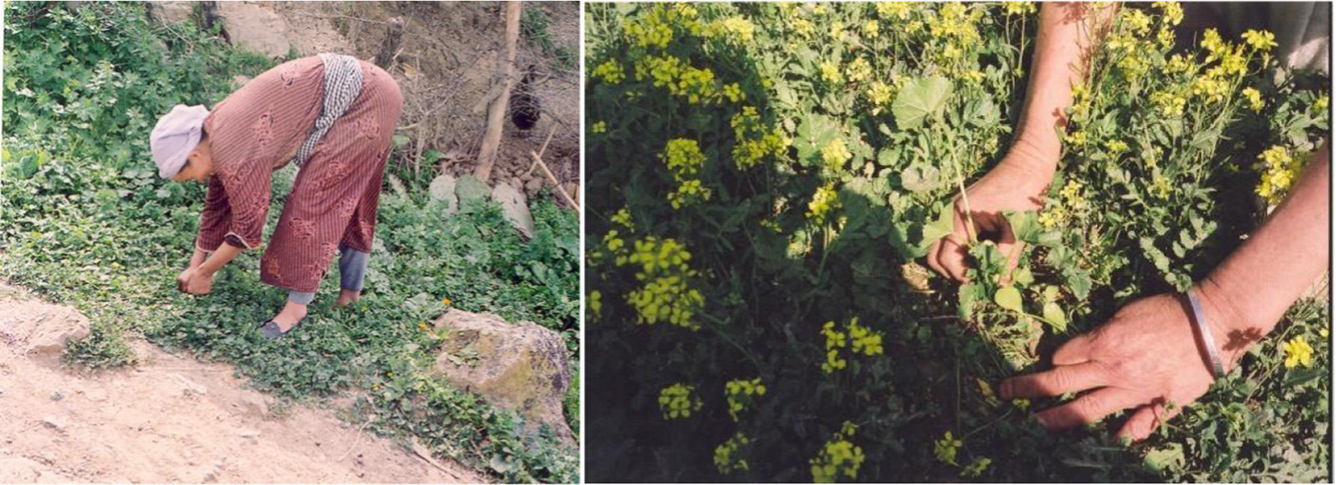
**Collecting WLVs (left: collection *Malva *** **spp. Izara, Taounate, right: *Malva *** **spp. in Sidi Sinoun, Taounate).**

Everywhere, women had very clear knowledge about specific preparation practices needed to make different species palatable (Figure 2). WLVs are most often used as a side dish (eaten with bread) or in couscous. Women readily explained their views on which species need to be mixed to achieve a good taste and texture, as well as the ratios of different species needed. However, opinions differ greatly on this topic, including from one neighbour to the next.

### Geographic variation in use and nomenclature

**Figure 3:**
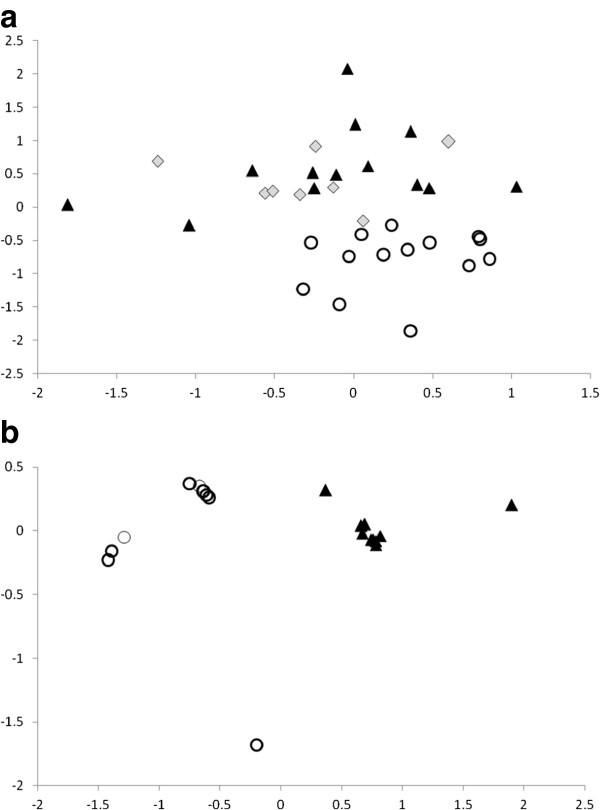
**MDS of free lists in different sites, demonstrating overlap between nomenclature / knowledge in villages using same market. a**. Three villages from Taounate region, Sidi Sinoun (black triangle, N=13), Izara (grey dimond, N=8), Chachia (open circle, N=14). **b**. Two villages from Azilal region, Habliss (black triangle, N=10), Ibiyane (open circle, N=10).

Significant differences in the salience (frequency with which they are reported in free lists and discussed by informants), diversity and nomenclature of species reported can be seen between the three regions of study, as well as between villages within regions (Table 2). In a study from Benin, Achigan-Dako and colleagues [34] similarly describe significant variation between phytogeographical regions, demonstrating that geography is a stronger determinant of traditional vegetable use than socio-linguistic group (especially for wild species). From our data, we were also able to identify significant variation in WLV knowledge within geographical regions, in addition to between regions. MDS graphs produced from free lists show the two dimensional relationship between informants’ “knowledge” in each province (Figure 3). In Azilal there is greater variation between villages than between informants within each village. In Taounate province, informants in Izara and Sidi Sinoun are intermixed (greater variation between informants than between villages), while informants from Chachia form a distinctive cluster. Because the villages are quite far apart and have very limited social ties, we believe that the overlap between Izara and Sidi Sinoun is due to knowledge transmission occurring in the weekly market that inhabitants of both villages visit regularly (the villages Ibiyane and Habliss in Azilal are geographically very close – closer than Sidi Sinoun and Izara, but use different markets). There was an exceptional diversity of WLV for sale in Ourtzagh market (used by Izara and Sidi Sinoun) relative to all the other markets surveyed. We believe that this may be an important site for horizontal knowledge transmission.

**Table 2:** Differences in species diversity and salience between villages (as determined by free listing data)

**Region**	**Taounate**	**Taounate**	**Taounate**	**Azilal**	**El Haouz**
**Village**	**Sidi Sinoun**	**Izara**	**Chachia**	**Habliss**	**Tassa Ouirgen**
Number of HH listed	22	23	19	27	apx.80
Number of FFQ conducted	22	21	18	18	0
Number of Free lists obtained	13	8	14	10	6
Average length of free list	6.5	5.6	4.6	4.1	5.3
Min and max length of free lists	4-12	2-8	2-8	3-6	4-8
Top 5 WLVs	HOUMIDA (*Rumex* sp.)	KARN KEBICHA (*Erodium* sp.)	HOUMIDA (*Rumex* sp.)	TIBI (*Malva* spp.)	WA’MSA (*Foeniculum* sp. or *Ferula communis*)
	BAKOLA HOURA (*Malva* spp.)	HOUMIDA (*Rumex* sp.)	RJEL FELOUS (*Chenopodium* sp, or *Glebionis coronaria*)	IBITZ (*Rumex* spp.)	GERNOUNCH (*Nasturtium officinale*)
	KARN KEBICHA (*Erodium* sp.)	BAKOLA HOURA (*Malva* spp*.*)	MESK AAZARA (*Erodium* sp.)	TIFILISHOUT (cf. *Rumex* sp.)	TIBI (*Malva* spp.)
	BOUHAMOU (*Diplotaxis*/*Sinapis*)	KHOBIZA (*Malva* spp*.*)	BAKOLA HOURA (*Malva* spp.)	TAGHDIOUT (*Scolymus hispanicus* or *Carduus* sp.)	TAGERLOUCHT (*Portulaca oleracea*)
	KHOBIZA (*Malva* spp.)	GERNINA (*Scolymus hispanicus*)	SELK (*Beta macrocarpa* or *Emex spinosa*)	*only 4 sp. listed by more than one person	TIFLICHOUT (cf *Rumex* sp.)

### The role of markets in knowledge (or culture) transmission

“Cultural transmission is the process of acquisition of behaviours, attitudes, or technology through imprinting, conditioning, imitation, active teaching and learning or combinations of these” [35]. Multiple types of transmission process have been identified including: vertical, oblique and horizontal. Vertical transmission occurs when a cultural trait or knowledge is transmitted from a parent (or grandparent) to a child; horizontal transmission is transmission between peers, or individuals in approximately the same generation [35]. Almost all human food preferences are acquired. The fact that it is difficult to condition food preferences (but not aversions) regardless of palatability, suggests that food preferences are heavily socio-culturally conditioned [36], presumably through the process of cultural transmission.

“Markets and other economic institutions do more than just allocate goods and services: they also influence the evolution of values, tastes and personalities” [37]. Markets and economies can affect the process of cultural transmission (the way in which we acquire values and desires) because economic interactions are personal, strategic and durable connections among people whose identities matter for the outcome [37]^a^. In other words, markets are an important site of cultural transmission, specifically horizontal cultural transmission, the form of transmission which is associated with rapid change in knowledge and knowledge systems [38]. Acerbi and Parisi [39] note that vertical knowledge transmission is more important in small-scale traditional societies whereas horizontal transmission plays a greater role as societies increase their economic activity.

The effect of economic institutions (trade) on plant knowledge and food plant knowledge has been described by Turner [40], and Turner and Loewen [41] in their work on plant nomenclature among indigenous groups on the North West Coast of North America. Their work showed that groups with historical trade relations shared greater similarity in plant names than groups which were geographically closer to each other but who had not traded with each other. Linguistic evidence supports the fact that plant knowledge and nomenclature were exchanged along trade networks in addition to plant material [41]. Conversely, in a context where markets seem not to be acting to facilitate knowledge transmission, Ogle and colleagues [9] note greater variation between villages than within villages in number, variety and  major species’ of WLVs consumed. The transmission of knowledge within trade networks or markets is distinctly different than cultural diffusion, in which horizontal transmission occurs between groups in close proximity to each other.

Of the 12 small and medium sized rural markets we worked in across Morocco, WLVs were found for sale in only four of them. Two of these markets were the largest markets in their respective areas, the other two were Ourtzagh, and the market adjacent to it, Galaz (the markets used by people from Sidi Sinoun and Izara). Preventing the loss of WLG knowledge in Morocco will require the maintenance of traditional agricultural systems, and the associated knowledge, and socio-cultural systems [10,42]. The maintenance of traditional market systems may be essential for the preservation of the complex traditional food and agricultural systems which they act to integrate. This is particularly true in a country with very high diversity of ecosystems, often within a small geographical area, and a long history of traditional market systems.

## Conclusions

Our data contribute to the evidence of a high diversity of WLVs used in Morocco. Research on WLVs has likely been historically limited by the strong social stigma some people ascribe to WLVs, a gender gap between trained botanists who are mainly male and the women who hold the knowledge of WLVs, and, as elsewhere in the world, high geographical variability in local names used. Importantly in Morocco, the very seasonable nature of use and flowering patterns of most WLV species, has made it very difficult to get a complete picture of all the species used, even in sites which have been visited over multiple years.

Our data show that at least in some regions WLVs were almost universally consumed when in season. This, combined with the fact that increased fruit and vegetable consumption is now believed to be one of the best predictors of a wide range of health outcomes [43,44], suggests an important potential for WLVs to contribute to the mitigation of both chronic nutrition-related disease and micronutrient deficiency (if their supply can be sustainably increased and expanded). As in other places, maintenance of knowledge for WLV identification and preparation will be necessary for their continued use [40,45]. Knowledge of WLVs in Morocco is clearly highly nuanced, very highly variable, and susceptible to rapid change. WLVs in Morocco provide an extremely interesting case study in which to further study the horizontal and vertical transmission of traditional or local knowledge. In Morocco, markets may be an important site where food preferences and choices are shaped through cultural transmission.

There is a great need for more research on WLVs in Morocco: nutrient composition, contribution to local diet and nutrition, as well as the potential of WLVs and other traditional foods to play a role in mitigation of the nutrition transition. We need to better understand if and how WLVs and other traditional foods can be incorporated into public health nutrition messages and food-based strategies to mitigate the double burden of nutrition Morocco now faces.

## Endnote

^a^Studies of food brand preferences show that personal contact is the most effective, and peers are most influential (Bowles [36]).

## Competing interests

The authors declare that they have no competing interests.

## Authors’ contributions

BP and AO conceived of the research idea. TJ and PB secured funds and supported study design. BP and AO collected the data. MIT and AO provided the botanical identification. BP and AO analysed the data. BP prepared the first draft of the paper. All authors contributed to the preparation of the final draft. All authors read and approved the final manuscript.
